# A Parametric Model of the Mitral Valve for Finite Element Patient-Specific Simulations

**DOI:** 10.3390/bioengineering13060676

**Published:** 2026-06-10

**Authors:** Alicia Menéndez Hurtado, Sergejus Borodinas

**Affiliations:** Department of Applied Mechanics, Vilnius Gediminas Technical University, Saulėtekio al. 11, 10224 Vilnius, Lithuania; sergejus.borodinas@vilniustech.lt

**Keywords:** finite element method, parametric model, mitral valve, fem

## Abstract

Finite element models of the mitral valve can be useful tools for physicians as a predictive tool for surgical planning, teaching, or observation. In order to seamlessly implement these tools in a clinical setting, the process for the creation of the models needs to take into account the diagnostic procedures and tools available to physicians. In this study, a rapid patient-specific model for clinical applications is developed, creating a parametric geometry from measurements routinely taken during the diagnostic process and maintaining a low computational cost through simplifications in material and boundary conditions. The healthy valve model is then validated against ultrasound images from peak diastole to peak systole, finding a good conformity despite simplifications. These results can serve as a stepping stone towards the development of a clinical digital twin of the mitral valve that combines engineering knowledge and medical process.

## 1. Introduction

Cardiovascular diseases are one of the leading causes of death worldwide, with valve-related diseases accounting for the highest morbidity and mortality rate worldwide. Mitral regurgitation, a disease of the mitral valve that causes blood to flow back into the atrium, is the most common valvular heart disease in Europe [1]. Due to its prevalence, efforts have been made to pave the way for physicians and recreate the geometry and mechanics of the mitral valve virtually.

The mitral valve is one of the four valves of the heart and separates the left atrium and ventricle, and it acts as a one-way valve that closes with the contraction of the ventricle. The mitral valve consists of the annulus, two leaflets, *chordae tendineae*, and papillary muscles. The annulus is a ring-shaped structure attached to the walls of the heart that contracts and relaxes with them. The leaflets are attached to the annulus and form the physical barrier that stops the blood when the valve closes. On the ventricular side, they are held in place by the *chordae tendineae*, tendinous strings that prevent the valve from billowing into the atrium by connecting it to the ventricular wall through the papillary muscles [2].

In recent years, many different finite element mitral valve models have been developed, the main focus being patient-specific models due to their potential medical use. In order to create these models, the geometry can be extracted using different imaging techniques, each offering varying degrees of complexity and anatomical precision [3,4]. Microtomography allows the extraction of a very detailed geometry, including the structure of the *chordae tendineae* [5], which cannot be seen with other imaging technologies. The geometry created through this technique can take into account variations in shape of the leaflets, asymmetries of the valve, and branching patterns of the *chordae tendineae*, making it a highly accurate geometric model. However, due to the intrinsic characteristics of microtomography, it is not possible to extract the mitral valve geometry from live humans. Regular computerised tomography can be used in live humans to extract geometry and is capable of producing precise patient-specific valve geometries [6,7], but it is an imaging technique that uses ionising radiation, which is not recommended for all patients. The preferred method for the extraction of geometry in the literature is cardiac ultrasound, which is the standard imaging technology used by physicians for the diagnosis and visualisation of the valvular structure. Therefore, it is most suitable for a patient-specific model without the need for additional time, risk, or discomfort for the patient that additional procedures would mean. In this category, models still follow various levels of complexity, in this case focusing on the addition of *chordae tendineae*, the complexity of boundary conditions, or material properties [8,9,10].

Mathematical models offer the option of studying generalised characteristics and structures through a simplified geometry following the shape of cylinders or truncated cones with slight variations [11]. Although highly simplified and theoretical, these models can serve as a basis for creating more anatomically accurate geometries [12]. All previous models discussed rely on different tracking processes to extract the valvular geometry into a usable FE geometry, a time consuming process that requires specialised knowledge of the software. To facilitate this process, efforts have been made to create parametric models of the mitral valve. Hong et al. [13] have created a parametric model using four primary landmarks for the shape of the annulus and a combination of sinusoidal functions for the free edge of leaflets to study the effect of *chordae tendineae* location. The model at peak diastole features a saddle-shaped annulus and straight leaflets. Alternatively, the model by Alleau et al. [14] is built with a flat and fixed annulus and compares a healthy and pathological case by simulating broken *chordae*. The toolbox created by de Oliveira et al. [15] gives a deeper insight into the parameters used for the model and offers dedicated software to build the geometry that can then be transformed into an STL file. However, these models do not consider the clinical process in their selection of parameters, using measurements that are not generally considered by physicians and that might not be available in routine imaging.

In this article, a simplified model of the mitral valve is created with the objective of future clinical implementation. For this purpose, the parameters have been selected from measurements routinely taken during ultrasound imaging and computational resources are kept as low as possible to allow future clinical implementation. Additionally, the geometry is validated from peak diastole to peak systole, considering the closing process itself.

## 2. Materials and Methods

### 2.1. Geometry Parameters

A parametric approach was chosen for the construction of this model. Seven parameters were selected based on the measurements routinely taken by physicians in diagnostic ultrasounds, making the acquisition of measurements quick and convenient.

**Figure 1:**
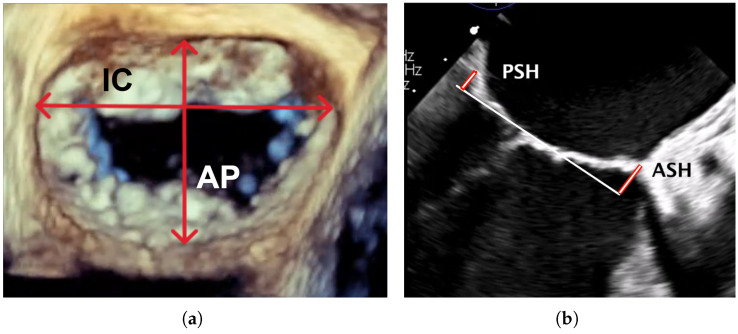
Parameters used to construct the geometry of the mitral valve annulus. (**a**) Anteroposterior (AP) and intercommissural (IC) distance measurements of the annulus from a surgeon’s view ultrasound image. (**b**) Anterior saddle height (ASH) and posterior saddle height (PSH) measurements (in red) of the annulus from an ultrasound image. The white line indicates the valvular plane.

Four parameters are used for the construction of the annulus shape, while the other three are used for the free edge and the leaflets. All measurements can be extracted directly from routine ultrasound imaging. In the case of the annulus, measurements for the intercommissural distance (IC) and the anteroposterior distance (AP) are taken during diastole, while the mitral valve is open, from the 3D ultrasound image of the valve in surgeon’s view, i.e., as seen from the atrium. The other two measurements are the anterior saddle height (ASH) and posterior saddle height (PSH), which indicate the curvature of the annulus. These measurements are shown in Figure 1.

**Figure 2:**
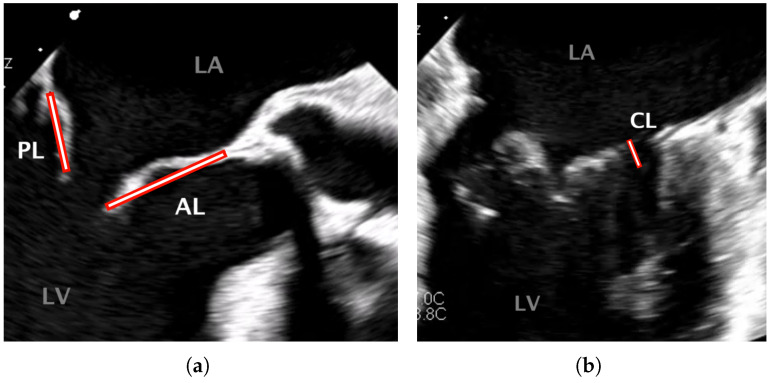
Parameters used to construct the geometry of the mitral valve leaflets, shown in an ultrasound image. LA: Left atrium. LV: Left ventricle. (**a**) Anterior leaflet (AL) and posterior leaflet (PL) length measurements. (**b**) Commissural length (CL) measurement.

The remaining parameters are used to build the leaflets and the general shape of the valve. The anterior leaflet length (AL) and posterior leaflet length (PL) are measured during diastole at a 120º angle, which shows the mid section of the valve where the maximum length of both leaflets can be seen. A 30º angle view shows the commissural length (CL) when the valve is open. These measurements are shown in Figure 2.

### 2.2. Parameter Data Acquisition

All of these parameters can be measured directly while taking ultrasound images, making the process fast and convenient for physicians. For this paper, the measurements were taken after the fact by author AMH, a non medically trained engineer, and checked by a trained professional for accuracy. In Table 1 the measurements obtained for this valve are compared with healthy ranges from the literature.

It is important to note that minor discrepancies can be expected, as even among healthy individuals there is a wide range of measurements that can be affected by age, size, and natural variability. Measurements taken from these ultrasounds can reasonably be assumed to be from a healthy individual, as confirmed by a cardiologist.

### 2.3. Geometric Considerations

**Figure 3:**
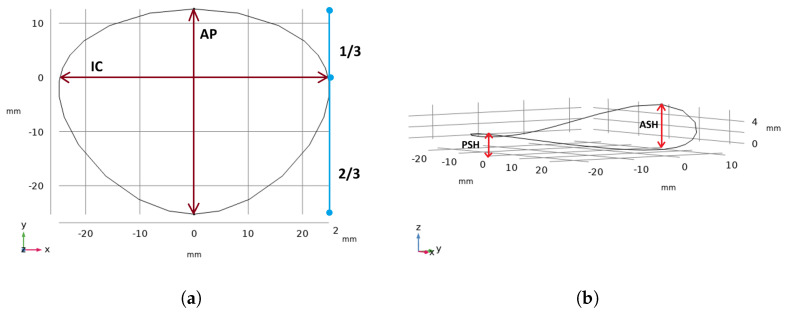
Parameters used in the annulus construction. (**a**) Surgeon’s view of the annulus with AP and IC parameters. (**b**) Three dimensional shape of the annulus with ASH and PSH parameters.

The characteristic saddle shape of the annulus is defined through the diameters of the AP and the IC, respecting the proportion of the shape of the annulus [18], where the IC sits at one third of the distance of the AP, as shown in Figure 3.

**Figure 4:**
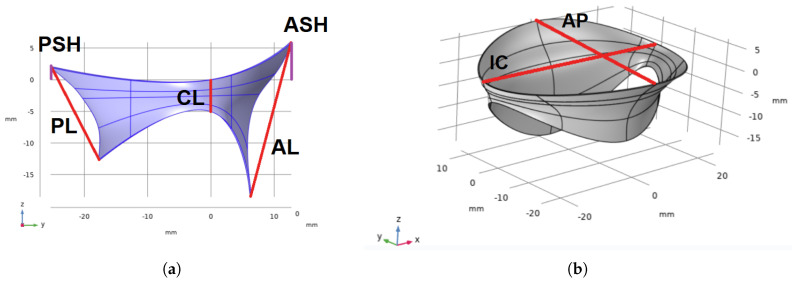
Parameters used in the full valve model. (**a**) Side view of the valve model with AL, PL, CL, ASH, and PSH parameters. (**b**) Isometric view of the model with AP and IC parameters.

Then a curve is built for the free edge of the leaflet. This curve takes into consideration the length of the leaflets and commissures, as well as the diameters of the annulus. Three additional curves are created between the annulus and the free edge, imitating the natural curvature of the relaxed mitral valve during diastole instead of producing straight leaflets at peak diastole. The final geometry of the model is shown in Figure 4.

### 2.4. Software

To build the geometry in the software COMSOL Multiphysics ® (v. 6.0. COMSOL AB, Stockholm, Sweden) from the parameters, each closed interpolation curve was individually defined through 4 points dependent on the selected parameters. All points are defined in Table 2.

These curves were then lofted to create the valve surface and the final shape of the geometry. The boundary conditions were applied to this surface. Additional vertical divisions have been created to assist in the application of the boundary conditions, but the shape of the geometry is unchanged by these.

### 2.5. Material Properties and Boundary Conditions

The mechanical behaviour of mitral valve leaflets has been experimentally studied *ex vivo*, finding that they follow anisotropic and hyperelastic models [19,20,21]. In this study, however, the leaflets were modelled as an isotropic linear elastic material with a variable Young’s modulus. This is a simplification of the known properties of the leaflets that has been proven to produce good results [22,23]. Additionally, studies by Krishnamurthy et al. [24] show that in vivo valves under physiological conditions follow a mostly linear elastic model behaviour.

The leaflets are modelled as an isotropic linear elastic material, with a variable Young’s modulus and a Poisson’s ratio of 0.45 [14]. The value of the Young’s modulus (E) is highest at 3 MPa in the central part of the leaflets, decreasing towards the commissures. The decrease in stiffness towards the commissures accounts for bigger deformations during the simulation, and therefore prevents points of stress that can cause a singularity in the model. The value of E in each position follows Equation (1), where *x* follows the direction of *IC*.

(1) $$E=3\frac{IC-|x|}{IC}\left[\mathrm{MPa}\right]$$

**Figure 5:**
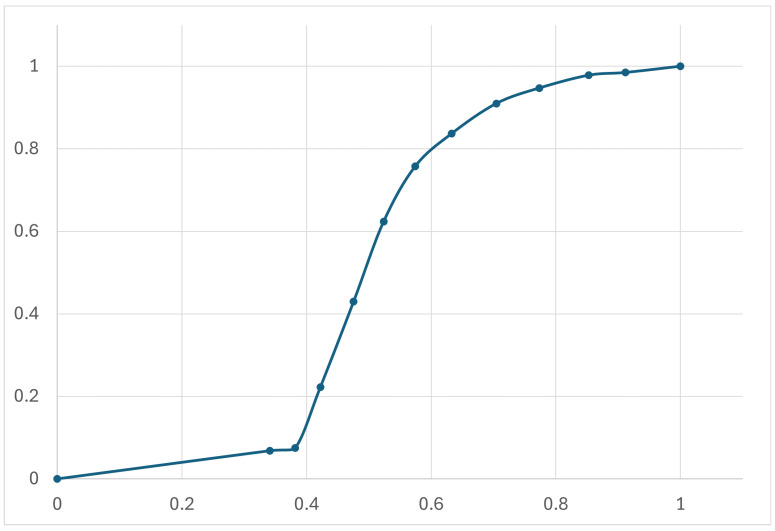
Function describing ventricular pressure during systole, defined as f(t).

The movement of the annulus decreases the distance between leaflets, increasing the coaptation area and improving the efficiency of the valve. This is simulated with a prescribed displacement of the anterior and posterior annulus sections, moving towards the atrium and center of the valve. For the anterior annulus section, the movement follows Equation (2):

(2) $$\left\{\begin{array}{c} u_{ant,y}\left(t\right)=-f\left(t\right)\cdot \frac{3\cdot ASH}{4}\\ u_{ant,z}\left(t\right)=f\left(t\right)\cdot \frac{ASH}{3} \end{array}\right.,$$

and the posterior annulus follows Equation (3):

(3) $$\left\{\begin{array}{c} u_{post,y}\left(t\right)=f\left(t\right)\cdot \frac{2\cdot ASH}{3}\\ u_{post,z}\left(t\right)=f\left(t\right)\cdot \frac{ASH}{2} \end{array}\right.,$$

where f(t) is defined as the ventricular pressure during the closing of the valve (systole), as shown in Figure 5. This function follows the shape of the ventricular pressure during closing, when the ventricle contracts and the increased pressure forces the valve to close. The points have been experimentally taken from an average cardiac cycle pressure plot, with a piecewise cubic interpolation.

This function is the base of all boundary conditions, as it drives the movement of the entire ventricle during the cardiac cycle, and therefore all valve movements follow the same shape. The function has been normalized to allow adjustment to the corresponding magnitude with each boundary condition.

**Figure 6:**
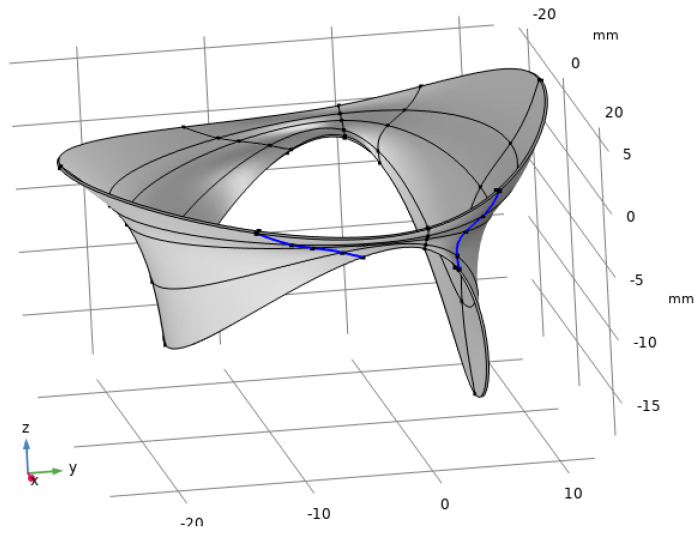
Anterolateral edges where displacement is applied to prevent rippling and ensure good contact.

In order to prevent a rippling of the leaflets caused by the geometry and the forces applied to it, an additional displacement of the commissures is added, adjusting the geometry to ensure good contact and prevent convergence issues. Equation (4) is applied symmetrically at both sides, indicated in blue for the anterolateral commissure in Figure 6.

(4) $$u_{anlat,x}\left(t\right)=3\cdot f\left(t\right)\left[\mathrm{mm}\right]$$

A load is applied to the face of the leaflets, pushing them towards the center of the valve and contributing to the characteristic curved shape of the leaflets during closing. Due to the geometrical features of the valve, the force is lowest at the centre and increases towards the commissures to ensure a good closure. The force applied to the anterior and posterior leaflets are defined by Equation (5) and Equation (6) respectively.

(5) $$\left\{\begin{array}{c} F_{ant,y}\left(t\right)=-5f\left(t\right)-3.5\frac{\left|x\right|}{IC}\cdot f\left(t\right)\;\;\left[N\right]\\ F_{ant,z}\left(t\right)=4f\left(t\right)+\frac{\left|x\right|}{IC}\cdot f\left(t\right)\;\;\left[N\right] \end{array}\right.,$$

(6) $$\left\{\begin{array}{c} F_{post,y}\left(t\right)=6f\left(t\right)+4\frac{\left|x\right|}{IC}\cdot f\left(t\right)\;\;\left[N\right]\\ F_{post,z}\left(t\right)=3.5f\left(t\right)+2\frac{\left|x\right|}{IC}\cdot f\left(t\right)\;\;\left[N\right] \end{array}\right..$$

The contribution of the *chordae tendineae* to the function of the valve is simulated by applying an edge load at the free edge of the valve. This load pulls the edge of the leaflet towards the apex of the ventricle, simulating the presence of chordae, which tether the leaflets and prevent them from billowing into the atrium. This contribution of CT is defined by Equation (7) and creates an evenly distributed load along the edge of the leaflets instead of discrete attachment points of individual *chordae tendineae*.

(7) $$\left\{\begin{array}{c} F_{CTant,z}\left(t\right)=-1.9f\left(t\right)\;\;\left[N\right]\\ F_{CTpost,z}\left(t\right)=-2.1f\left(t\right)\;\;\left[N\right] \end{array}\right..$$

### 2.6. Contact

The internal side of both leaflets was defined as a contact pair to achieve the final closed shape. From the different contact models available, Nitsche’s model offered the best stability and convergence during the simulation. This model has three possible variants, depending on the value of θ in Equation (8).

(8) $$\delta u-map\left(\delta u\right)=-\frac{1}{p_{n}}\left[p_{n}\delta u-p_{n}map\left(\delta u\right)-\theta \delta T_{a}\right]+\frac{\theta }{p_{n}}\delta T_{a},$$

where $T_{a}$ is the nominal traction vector, $p_{n}$ is the penalty factor and θ controls the symmetry of the formulation.

If θ=1, the formulation is symmetric, offering the most consistent formulation given a suitable choice of $p_{n}$.

If θ=−1, the formulation is skew-symmetric, which is less sensitive to $p_{n}$.

If θ=0, the formulation is incomplete. This formulation is less sensitive to $p_{n}$ than the symmetric option, but not as robust as the skew-symmetric. This was the formulation used for this model, with a penalty factor $p_{n}=1$.

When using Nitsche’s method, the penalty factor $p_{n}$ is considered a stabilization factor, unlike in the penalty formulation, where it acts as a spring stiffness [25].

### 2.7. Mesh

A tetrahedral mesh is used for the model, with the metrics taken from COMSOL Multiphysics 6.3: tetrahedral elements-15862, triangles-11240, edge elements-1697, vertex elements-80. A mesh refinement check with three times the elements showed similar results, indicating adequate resolution of the mesh.

## 3. Results and Validation

**Figure 7:**
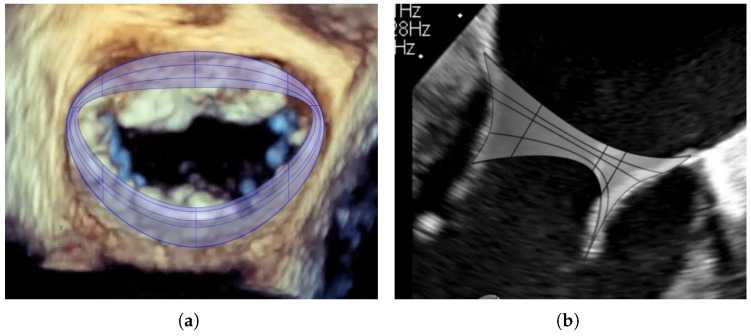
Comparison of the geometry at $t_{0}=0$ s with the ultrasound images. (**a**) Annulus geometry compared with a surgeon’s view ultrasound image. (**b**) Leaflet shape and annulus distance geometry compared with an ultrasound image.

This section aims to compare the results of the simulation with the ultrasound images taken from a healthy patient, both during systole and diastole and along the process of closing the valve. During diastole, the starting geometry is compared to the ultrasounds, as shown in Figure 7. The model conforms to the general shape of the annulus as well as the leaflets before forces or displacements are applied.

**Figure 8:**
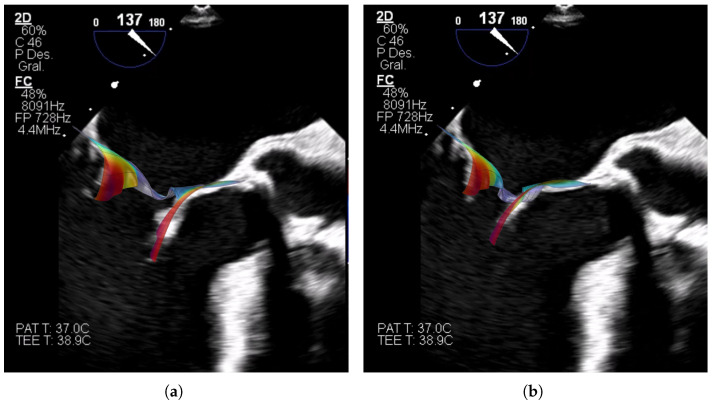
Comparison of the leaflet shape and annulus distance geometry with the ultrasound images at two points during closing. (**a**) At $t_{0.45}=0.09$ s. (**b**) At $t_{0.5}=0.1$ s.

**Figure 9:**
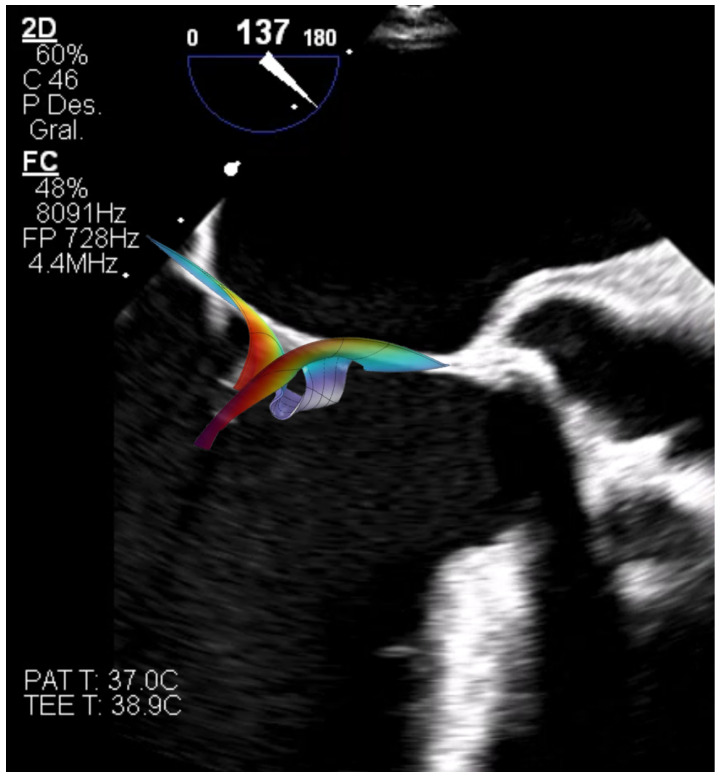
Comparison of the geometry at $t_{0.45}=0.2$ s, when the valve is fully closed.

During the closing process, two more frames are validated against the ultrasound images, as shown in Figure 8. Due to the temporal resolution of the ultrasound and the speed of the movement, which is completed in about 0.2 s, a total of only four frames were able to be used for the validation. The first frame shows the relaxed valve, at t = 0 s, as shown in Figure 7. The second frame is taken at t = 0.09 s, and the third at t = 0.1 s, as shown in Figure 8. The last frame compares the model at peak systole, when the valve is fully closed, as shown in Figure 9.

The simulation run for 1 h and 13 m.

## 4. Discussion

The work presented in this manuscript aims to contribute to the field of finite element models of the mitral valve, with a focus on patient-specific models that have the potential to be used in a clinical setting. For this reason, parameters that are obtained routinely through the diagnostic process are selected. In order to maintain a low requirement of computational resources and time, simplifications are made for the geometry and boundary conditions, attempting to strike a balance between speed and accuracy of the results. The geometry at peak diastole can be visualised instantly and differences in annulus shape or leaflet size can be immediately observed in 3D. After the simulation, the valve model can be analysed throughout systole from different angles, observing the movement of the leaflets and the annulus from peak diastole to peak systole, a movement that takes 0.2 s of the cardiac cycle to complete.

**Figure 10:**
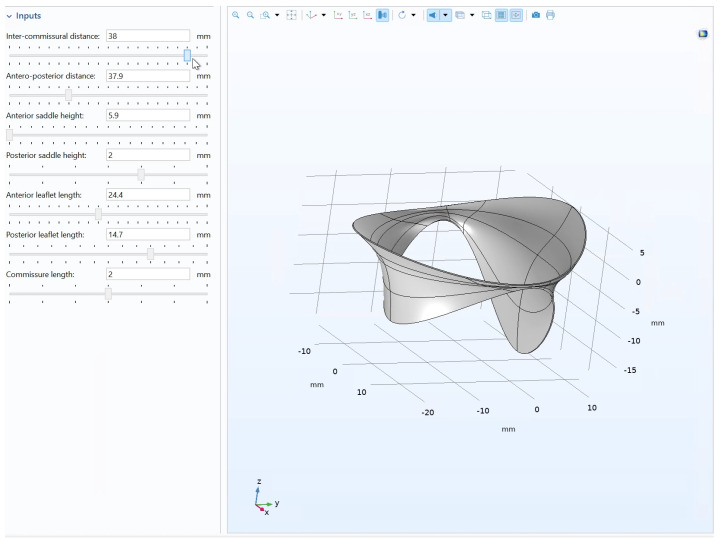
Input fields in the current app, where the geometry can instantly be changed and adapted.

Comparison of simulation results with ultrasound images shows that a good compromise between computational efficiency and accuracy of the finite element model has been reached. The computational cost is low, as the simulation can be run in an average clinic computer without significant delays. The current setup is built as an application through COMSOL itself, where relevant measurements can be input as shown in Figure 10, with the future development of a digital twin for a more simplified and fast user interface.

However, as a simplified model, it has limitations that must be taken into account.

### Limitations and Further Work

The simplified geometry allows for an instant visualization of the shape and characteristics of the valve during peak diastole, but the geometry obtained is always symmetric and does not take into consideration minor anomalies or deviations. Additionally, the commissures during the simulation do not adjust to anatomical features, instead being used to compensate for the deformation and movement of the annulus. The simplicity of the geometrical model construction allows for the simulation of some diseased valves, such as a flattened or calcified annulus, while further research is necessary to expand the model to other common valvular diseases, such as leaflet prolapse.

The leaflets are modelled as linear elastic material despite their known behaviour as a hyperelastic material. This simplification was based on previous models [22,23] that showed good results. Although a hyperelastic model is undoubtedly closer to an anatomical valve, it requires longer to simulate and higher computational resources. A deliberate choice was made to use an isotropic linear elastic material to simplify the model and reduce simulation time. For similar reasons, the *chordae tendineae* were simplified as an edge load instead of discrete strings. This approach has been used in the literature [26] and it has been verified that marginal *chordae tendineae* can be implicitly modelled as boundary conditions to the leaflets free margin [27]. However, this simplification can still cause major differences in discretely modelled *chordae tendineae*, particularly when studying stress distribution or diseases that specifically target the subvalvular system.

The model has been validated only through qualitative measures, as quantitative metrics would require additional manual segmentation or contour extraction that would introduce additional simplifications and uncertainties. A quantitative validation will be carried out in future models, when comparison between healthy and diseased simulations is available.

This model serves as a proof of concept investigation, as so far it has only been tested with one patient due to the limited availability of imaging data provided by the collaborating clinical team. A more in depth analysis is on the works for a wider comparison of models and geometries, including diseased valves.

## Figures and Tables

**Table 1:** Parameter measurements taken for the valve model and healthy ranges for adults taken from the literature.

Parameter	Healthy Range (mm)	Measured (mm)
Anteroposterior length (AP)	35.4±3.9 ^1^	37.9
Intercommissural length (IC)	40.1±4.8 ^1^	49.5
Anterior leaflet length (AL)	23.4±2.9 ^2^	24.4
Posterior leaflet length (PL)	13.8±2.9 ^2^	14.7
Commissural length (CL)	8.7±2.4 ^2^	5
Anterior saddle height (ASH)	11.7±3.9 ^1^	5.9
Posterior saddle height (PSH)	−	2

^1^ [16] ^2^ [17].

**Table 2:** Points defining each curve for the construction of the parametric geometry.

	X Axis (mm)	Y Axis (mm)	Z Axis (mm)
Annulus curve	0	AP/3	ASH
	IC/2	0	0
	0	−2·AP/3	PSH
	−IC/2	0	0
Middle top curve	0	0.8AP/3	ASH−0.6·AL/3
	0.98·IC/2	0	0.6·CL/2
	0	−0.85·2·AP/3	−PL/3+PSH·1.8
	−(IC/2)·0.98	0	−CL/2·0.6
Middle curve	0	0.6AP/3	ASH−AL/3
	0.95·IC/2	0	−CL/2
	0	−0.8·2·AP/3	−PL/3+PSH
	−0.95·IC/2	0	−CL/2
Middle bottom curve	0	0.45AP/3	(−AL+ASH)·0.6
	0.92·IC/2	0	−0.8·CL
	0	−0.7·2·AP/3	(−PL+PSH)·0.6
	−0.92·IC/2	0	−0.8·CL
Free edge curve	0	0.5AP/3	−AL+ASH
	0.9·IC/2	0	−CL
	0	−0.7·2·AP/3	−PL+PSH
	−0.9·IC/2	0	−CL

## Data Availability

The original contributions presented in the study are included in the article, further inquiries can be directed to the corresponding author.

## Abbreviations

The following abbreviations are used in this manuscript:

AL	Anterior leaflet length
AP	Anteroposterior
ASH	Anterior saddle height
CL	Commissural length
IC	Intercommissural
PL	Posterior leaflet length
PSH	Posterior saddle height
